# 

**Published:** 1898-09-10

**Authors:** 


					coooa T m JOL /M^ cocoa
IPV/ ABSOLUTELY PURE. ? DRUGS OR ALKALIES USED,
cC%/
Lascow Western Infirmary ?  3S=? ^ ^~*hukF.uLit am a NOR WICH HOSP.LTAL
No. 624. Vol. XXIV. GXSSkS Saturday,
Sept. 10th, 1898.
L soo p.404 j PRICE TWOPENCE.

				

